# A Small Subset of *Fruitless* Subesophageal Neurons Modulate Early Courtship in *Drosophila*

**DOI:** 10.1371/journal.pone.0095472

**Published:** 2014-04-16

**Authors:** David H. Tran, Geoffrey W. Meissner, Rachael L. French, Bruce S. Baker

**Affiliations:** 1 Department of Biological Sciences, San Jose State University. San Jose, California, United States of America; 2 Janelia Farm Research Campus, HHMI, Ashburn, Virginia, United States of America; AgroParisTech, France

## Abstract

We show that a small subset of two to six subesophageal neurons, expressing the male products of the male courtship master regulator gene products *fruitless^Male^* (*fru^M^*), are required in the early stages of the *Drosophila melanogaster* male courtship behavioral program. Loss of *fru^M^* expression or inhibition of synaptic transmission in these *fru^M^*(+) neurons results in delayed courtship initiation and a failure to progress to copulation primarily under visually-deficient conditions. We identify a *fru^M^*-dependent sexually dimorphic arborization in the tritocerebrum made by two of these neurons. Furthermore, these SOG neurons extend descending projections to the thorax and abdominal ganglia. These anatomical and functional characteristics place these neurons in the position to integrate gustatory and higher-order signals in order to properly initiate and progress through early courtship.

## Introduction

*Drosophila melanogaster* courtship is a multi-step, progressive series of behaviors established by sex-specific genetic and neurobiological components [1]–[4]. Work by our lab and others demonstrated that the expression of male products of the gene *fruitless* (*fru^M^*) is both necessary and sufficient to specify the potential for male courtship behavior. Approximately 2000 neurons in the central nervous system (roughly 2% of the CNS neuronal population) express Fru^M^ in clusters scattered throughout the central (CNS) and peripheral nervous systems [5]–[7]. In the periphery, expression was detected in subsets of primary sensory neurons of the sensory modalities implicated in courtship. Strikingly, *fru^M^*(+) neurons are dedicated to courtship as inactivating them disrupts courtship behaviors, but has no discernible effect on non-sexual behaviors.

Both *fruitless^Male^* and another component of the sex determination pathway, *doublesex^Male^* are involved in establishment of sexually dimorphic neural circuitry [8]. Activity of Fru^M^ is required for the survival of several male-specific neurons or sexually dimorphic projection patterns [9].

Multiple female sensory cues combine to regulate the activation and performance of male courtship behaviors. A feature of these multi-modal sensory inputs is the partial redundancy of some modalities: loss of visual, olfactory, or gustatory perception does not block courtship from occurring *Dros. melanogaster* [10]–[12]. Instead loss of any one of these three modalities delays the initiation and decreases the quantity of courtship. These functional redundancies suggest a compensatory integration of these multiple pathways in the courtship circuitry.

Several areas of the CNS have been identified as regions of higher-order processing and integration in the courtship circuitry [13]–[19]. *fru^M^(+)* projections densely innervate several regions: the lateral protocerebral complex, the mushroom bodies, the mesothoracic triangle in the ventral nerve cord, and the tritocerebral loop. Neurons projecting to the lateral protocerebral complex and mesothoracic triangle induce wing song behavior; subsets of these neurons require Fru^M^ and Dsx^M^ for survival in males [9]. The mushroom bodies are well-characterized regions controlling memory and learning.

One area of interest is the tritocerebral loop–which lies just ventral to the subesophageal ganglion (SOG)– an area of dense innervations targeted by gustatory, protocerebral/neurosecretory, and stomatogastric inputs [20]. Peripheral gustatory axons, from the mouthparts, subsets of the labellum, and stomatogastric nerves, target the tritocerebrum. The termini of descending tracts from the medial superior protocerebrum–notably the pars interecerebralis, a neurosecretory center–innervate the dorsal tritocerebrum. The higher-order interneurons that process and regulate gustatory inputs have not been fully characterized; the tritocerebral loop innervations likely integrate chemosensory and protocerebral inputs.

Here we targeted subpopulations of *fru^M^*(+) neurons that regulate chemosensory-dependent courtship initiation. We screened 72 *P[GawB] insertions*, driving an RNAi construct targeting *fru^M^*, *UAS-fru^M^*IR [21], for courtship defects that appear only under conditions where melanogaster is visually deficient [10]. The *P[GawB]4-57* line exhibited very limited overlap with *fru^M^*(+) neurons. *P[GawB]4-57* mainly overlapped with two to six *fru^M^*(+) neurons in the subesophageal ganglion (SOG), two clusters in the ventral nerve cord (VNC), and inconsistently an area just medial to the antennal lobe (mAL). Knockdown of *fru^M^* or inhibition of synaptic fusion limited to the SOG neurons resulted in infrared-specific courtship delays, and a failure to progress to copulatory behaviors. Strikingly, the tritocerebral projections of these neurons were significantly more extensive in males than in females; this male-specific projection pattern required Fru^M^ expression. These *fru^M^*(+) SOG neurons likely integrate chemosensory inputs in the tritocerebrum to modulate the initiation and progression of courtship.

## Results

**Figure 1:**
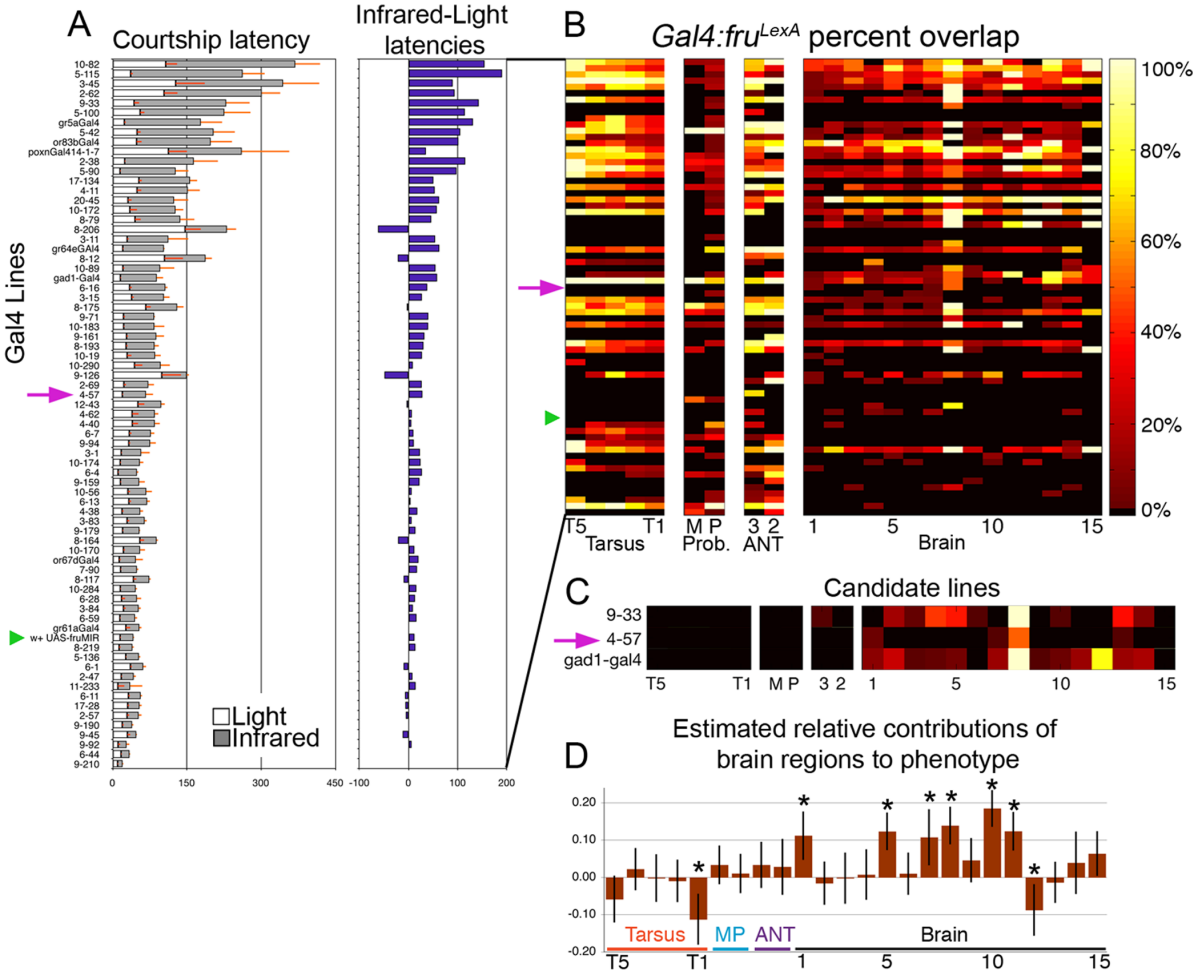
Mapping tissue-specific *fru^M^* repression to courtship latency phenotypes. Courtship latencies of *P[GawB] UAS-fru^M^IR* latencies depicted as stacked bars. A) White bars represent latencies in ambient light, while dark gray bars represent latencies in infrared (n = 10–44 males per genotype). Purple bars indicate the difference between dark-light latencies. Green triangles indicate position of control *UAS-fru^M^IR*/+ line. Purple arrows indicate position of *P[GawB]4-57 UAS-fru^M^IR*. B) Colored heat map representing the percent of *fru^P1-LexA^*(+) neurons in a given hemisphere cluster or peripheral segment that also express Gal4. Rows are *P[GawB]* lines in the same respective order as panel A. Columns represent the *fru^P1-LexA^*(+) clusters in foreleg tarsal segments (T5-T1), maxillary palp (M), labellum (P), 3^rd^ and 2^nd^ antennal segments (3, 2), and brain clusters 1–15, nomenclature from 21. C) Three lines that exhibited no or little peripheral Gal4 expression and limited central overlap with *fru^P1-LexA^*. D) Estimated relative contributions of each *fru^M^*(+) cluster to delays in courtship latency in infrared; values are coefficients of a linear regression derived from partial least squares analysis (see Methods). Error bars represent 95% confidence intervals estimated by resampling.

We identified subpopulations of *fru^M^*(+) neurons involved in chemosensory-specific pathways via a behavioral screen for proper courtship initiation in visually-deficient conditions. To do this we built on the findings of Meissner *et al*. [21] who screened a collection of approximately 1000 Gal4 P-element, *P[GawB],* insertions driving expression of two copies of a *fru^M^* RNAi construct, *UAS-fru^M^IR* (one insertion on the 2^nd^ and one insertion on the third chromosomes). We screened 65 *P[GawB]* and 7 candidate Gal4 lines for significant courtship delays to the first unilateral wing extension (courtship latency) in ambient and infrared light (Figure 1A). Due to lower visual resolution in infrared, we could not reliably identify the first instance of orientation/pursuit, the traditional method of measuring courtship latency. Henceforth courtship latency will refer to the average time to first unilateral wing extension.

We targeted lines with infrared-dependent courtship delays and CNS-specific, limited Gal4:fru^LexA^ overlap (Figure 1B) in order to identify candidate *fru^M^*(+) interneurons that regulate the activation of courtship. *fru^LexA^* is a knock-in insertion of the LexA transcriptional activator that drives expression of LexaOp-GFPnls in *fru^M^*(+) cells [21]. Three lines matched those criteria (Figure 1C). One line, *P[GawB]4-57*, drove Gal4 expression that overlapped *fru^LexA^*(+) neurons in only four *fru^M^*(+) cell clusters, clusters 1, 7, 8, and 13 in Figure C (Cluster nomenclature from [5]) with extensive overlap in the SOG cluster, cluster 8, in particular. A global principle-components-based regression analysis (Figure 1D)–using the entirety of the behavioral and expression data–correlated courtship latency delays with Gal4:fru^LexA^ overlap in several *fru^M^*(+) cell clusters (clusters 1, 5, 7–8, 10–11, 12), highlighting the importance of the *P[GawB]4-57*(+), *fru^M^*(+) clusters.

In addition, previous courtship assays had failed to detect male-male courtship, copulation, or aggression defects in *P[GawB]4-57* *UAS-fru^M^IR* males [21], suggesting that the *P[GawB]4-57*(+), *fru^M^*(+) neurons primarily functioned to regulate courtship initiation. Furthermore, the basal activity of *P[GawB]4-57* *UAS-fru^M^IR* males (0.48±0.1 line crossing/min. n = 24) did not significantly differ (p>.05) from wild type (0.46±0.1 line crossings/min, n = 24).

### *P[GawB]4-57*-driven *fru^M^*-targeted RNAi Leads to Courtship Delays in Infrared

**Figure 2:**
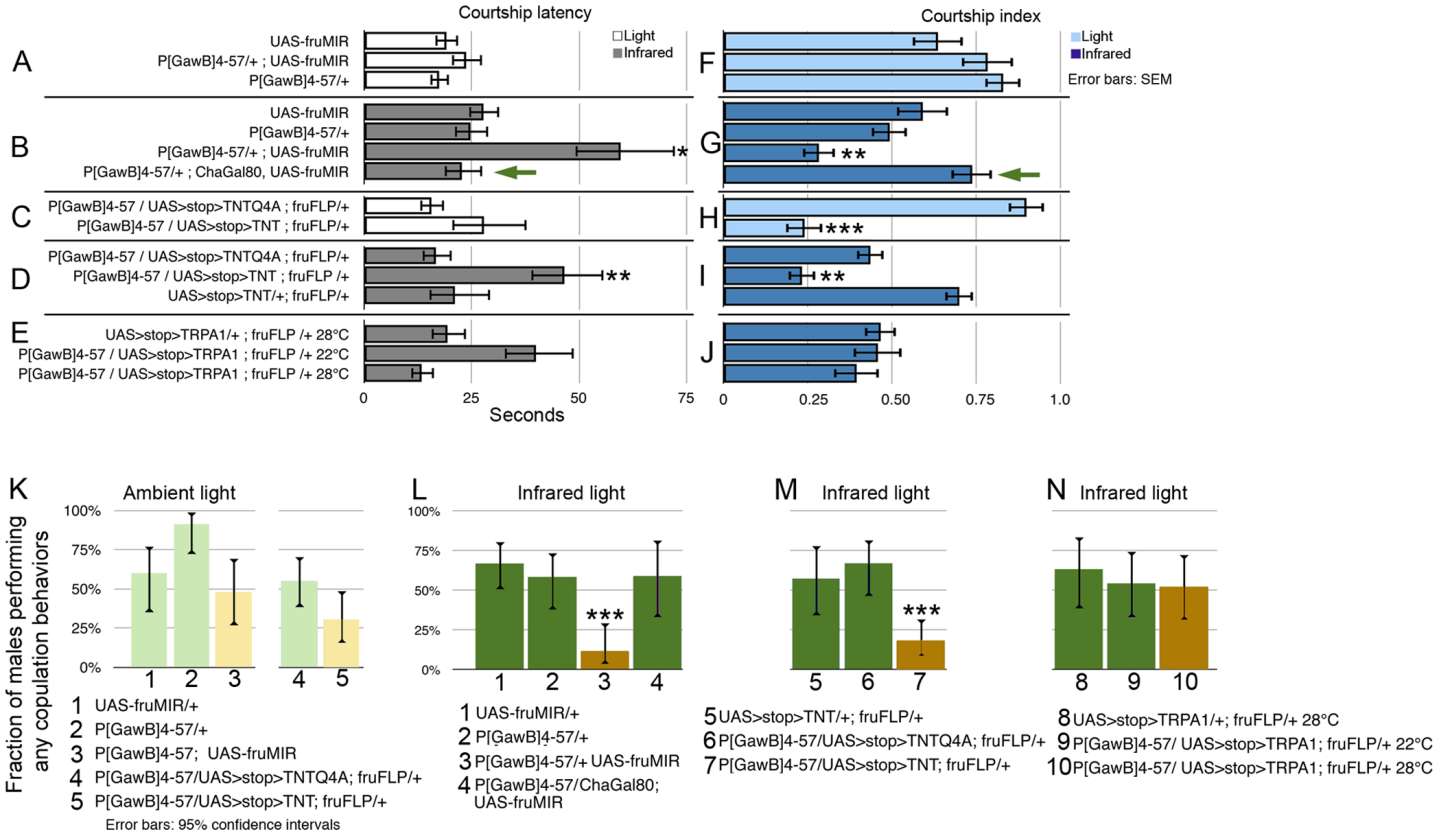
Tissue-specific knockdown of *fru^M^* expression or silencing synaptic transmission in *P[GawB]4-57*∩*fru^M^* neurons lengthened courtship latency, reduced courtship performance, and prevented progression to copulation. A–E) Horizontal bars indicate average time to the first unilateral wing extension by males in 10 minutes. F–J) Blue bars represent courtship index, measured as proportion of time spent performing courtship behaviors for 2 minutes after initiation of courtship. K–N) Vertical bars represent the fraction of males that attempt any copulation behaviors in 15 minutes. Darker bars represent courtship in infrared light versus ambient light. B) Under infrared light, *P[GawB]4-57*-driven *UAS-fru^M^*IR expression significantly lengthened courtship latency, unless *Cha-Gal80* (green arrow) was present; G) reduced courtship index; and L) led to most males failing to progress to copulation behaviors. D) Silencing *fru^FLP^*∩4-57 neurons via tetanus toxin (TNT) resulted in lengthened infrared courtship latency, H–I) reduced courtship index regardless of light conditions, and M) prevented males from exhibiting copulatory behaviors under infrared. E, J, N) Activation of *fru^FLP^*∩4-57 neurons, using UAS>stop>TRPA1 did not result in abnormal courtship. * =  p<.05, ** = p<.01, and *** = p<.001. Samples ranged from 23–44 males for latency, 11–28 for courtship index, and 17–44 for copulation.

Only under infrared light did we detect a courtship latency defect using the *P[GawB]4-57* driver (Figure 2A–B). *P[GawB]4-57* *UAS-fru^M^IR* males exhibited a two-fold increased delay in courtship initiation (58.9 secs., 95% confidence interval:40.4-87.9 secs., n = 17) compared to *UAS-fru^M^IR*/+ (27.5 secs., 95% c. interval: 21.4–36.0 secs., n = 38, p = .02) and *P[GawB]4-57*/+ (24.5 secs., 95% c. interval: 17.9–34.1 secs., n = 27, p = .006) controls. The overall quantity of courtship, denoted by courtship index, was also significantly reduced after courtship commenced (Figure 2F–G), as measured by the fraction of time devoted to courtship displays during the two minutes after the initiation of orientation/pursuit. *P[GawB]4-57* *UAS-fru^M^IR* males exhibited a significantly lower index (0.28±.05) compared to *UAS-fru^M^IR* and *P[GawB]4-57* controls (0.59±.08 and 0.49±.05, respectively, p<.004).

### *P[GawB]4-57*-driven Tetanus Toxin also Delays Courtship

We also wanted to determine if repression of synaptic transmission in *P[GawB]4-57*(+), *fru^M^*(+) neurons recapitulated the *fru^M^* RNAi results. Due to extensive non-Fru^M^ expression with the *P[GawB]4-57* driver, we used an intersectional strategy to inhibit synaptic transmission only in *fru^M^*(+), *P[GawB]4-57*(+) neurons FLP recombinase, under endogenous *fru^M^* regulation, limited the expression of tetanus toxin (TNT) to *fru^M^*(+), *P[GawB]4-57*(+) neurons, which we will denote as *fru^FLP^*∩4-57 [13]. TNT-mediated synaptic inhibition in *fru^FLP^*∩4-57 males (Figure 2C–D) significantly delayed wing extension behavior (46.55 secs., 95% interval: 31.2–69.4 secs., n = 26) only under infrared light compared to the inactive TNT^Q4A^ control (16.7 secs., 95% interval: 10.6–26.3 secs., n = 25, p = .001) or the *UAS>stop>TNT*/+; *fru^FLP^*/+ background control (21.1 secs., 95% interval: 13.3–33.4 secs., n = 17, p = .03). This behavioral delay was similar to that seen with *P[GawB]4-57* -driven *UAS-fru^M^IR* expression. Under ambient light, TNT expression in *fru^FLP^*∩4-57 cells (n = 23) did not significantly alter courtship latency compared to the control line (n = 21, Figure 2C).

When we quantified the courtship index (Figure 2H–I), expression of TNT in *fru^FLP^*∩*P[GawB]4-57* neurons also significantly reduced the quantity of courtship in both ambient and infrared light. Under ambient light, TNT expression depressed the courtship index to 0.24±0.1 compared to 0.90±.05 in the inactive control (p<.0001); under infrared, TNT expression led to a courtship index of 0.23±0.1 compared to 0.43±0.1 in the inactive control (p<.003) and 0.84±5.0 in the background control (p<.0001).

### *P[GawB]4-57*(+), Fru^M^(+) Neurons also Function to Initiate Copulation

Another aspect of courtship was also dependent on the proper function of *P[GawB]4-57*(+), Fru^M^(+) neurons (Figure 2K–N). Most *P[GawB]4-57*; *UAS-fru^M^IR* and *P[GawB]4-57/UAS>stop>TNT*; *fru^FLP^* males failed to exhibit any copulatory behaviors when visually deprived (compare Figures 2L–M to 2K). The majority of these males performed wing extension/song behaviors but made no detectable attempts at copulation, measured as at least one instance of pronounced curling of the abdomen (>90° curl from horizontal) and/or mounting attempts.

Under infrared-only lighting (Figure 2L), only 11.5% of *P[GawB]4-57*; *UAS-fru^M^IR* males (n = 30–42) progressed to any copulatory behaviors compared to 66.7% and 58.2% of *P[GawB]^4-57^*/+ and *UAS-fru^M^IR*/+ controls, respectively (p<.0001, Fisher’s exact test, n = 23–42). A similar defect was seen using tetanus toxin in *fru^FLP^*∩4-57 neurons (Figure 2M). Expression of TNT in *fru^FLP^*∩4-57 neurons resulted in only 18.2% of males exhibiting any copulation behaviors under infrared light (p<.0001, n = 44) compared to 66.7% of the inactive TNT controls (n = 21) or 57.4% (n = 21) of the background controls. Under ambient light, there was no significant difference between the genotypes for either behavior (Figure 2K).

Noting the effect of silencing synaptic transmission on the *fru^FLP^*∩4-57 neurons, we assayed the effect of depolarization via transgenic manipulation. The *UAS>stop>TRPA1* insert, encoding a temperature-sensitive cation channel [10], [24] allowed us to depolarize the *fru^FLP^*∩4-57 neurons by incubation at 28°-32°C (Figure 2E, J, N). Courtship latency, courtship index, and fraction of males performing courtship did not differ significantly from controls. Unilateral wing extension or abdominal curling was not induced in solitary males. These results suggest that activity from the *fru^FLP^*∩4-57 neurons, by itself, may not induce wing extension or copulation behaviors.

### *Cha-Gal80*-mediated Rescue of *P[GawB]4-57*(+) *UAS-fru^M^IR* Courtship Defect

**Figure 3:**
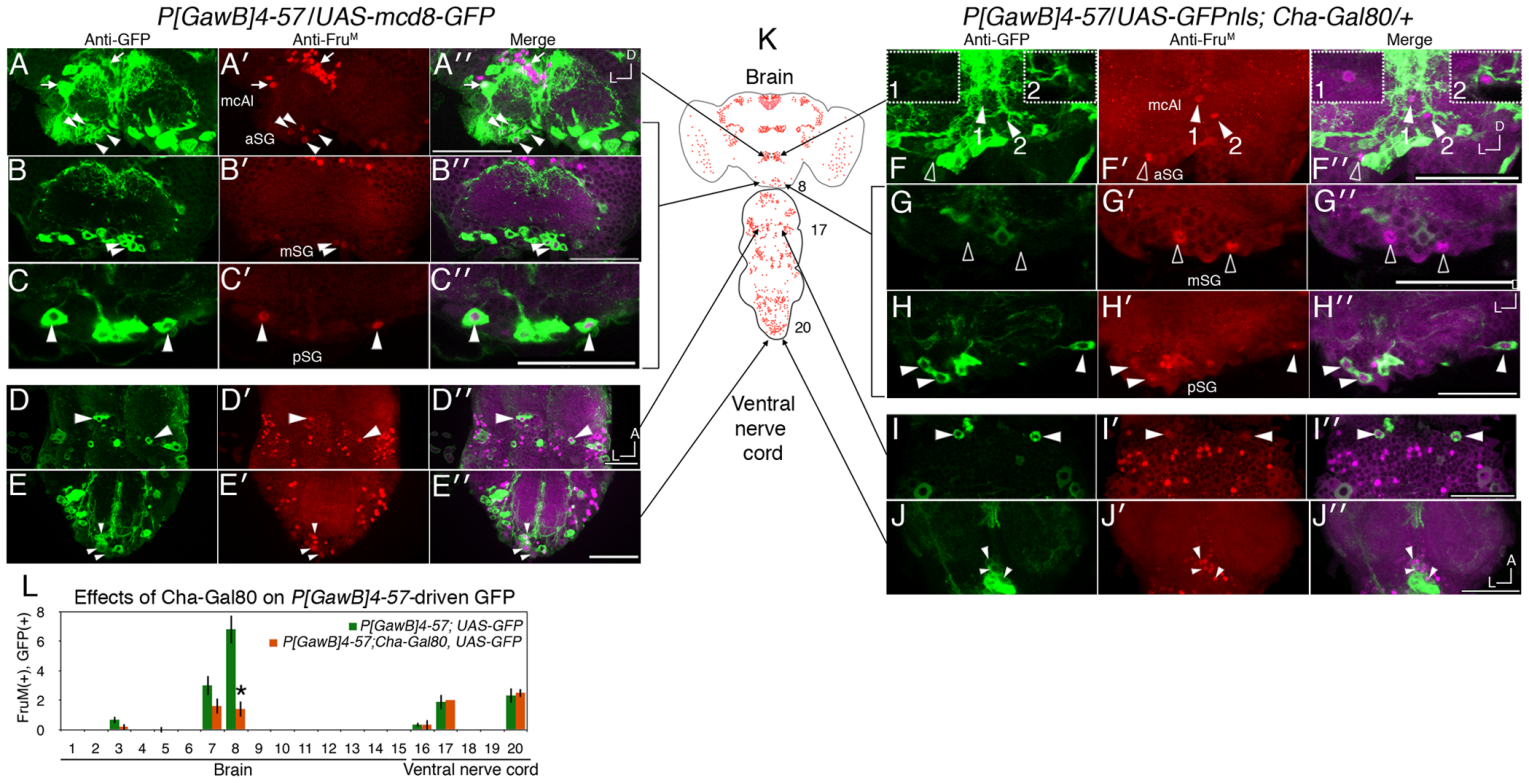
*Cha*-driven Gal80 inhibits *P[GawB]4-57*-driven Gal4 activity mainly in *fru^M^* SOG neurons. A–E) Partial Z projections showing GFP expression in *P[GawB]*4-57/*UAS-mCD8-GFP* expression compared to F–J) GFP expression in the presence of *ChaGal80/+*. Panels show confocal images of anti-GFP, anti-Fru^M^ (panels marked by ′), and merged fluorescence (marked by ′′). A) Two GFP(+), Fru^M^(+) were detected in the mcAl/DT6 cluster (arrows). Two to four smaller somas are found at 5–30 µm in depth, designated aSG (arrowheads). B) Two larger somas are found from 15–60 µm in depth, designated mSG. C) Two-three large somas are found at 60–100 µm in depth, designated pSG. D) Two GFP(+), Fru^M^(+) somas are found near the prothoracic/metathoracic boundary of the ventral nerve cord. E) Three to four GFP(+), Fru^M^(+) somas are located in the abdominal ganglion. F) The two mcAl/DT6 somas showed reduced, but detectable GFP expression. Insets show 2 µm Z-slices that highlight the mcAl/DT6 GFP(+), *ChaGal80*(+), Fru^M^(+) cells. F–G) *Cha-Gal80* repressed GFP fluorescence in all mSG neurons and a subset of aSG cells (hollow arroweads). Hollow arrowheads point to GFP(-), Fru^M^(+) cells, indicative of Cha-Gal80 repression, while filled arrowheads point to GFP(+), Fru^M^(+) cells. H–J) Fru^M^(+) pSG and ventral nerve cord cells still expressed GFP. K) Locations of imaged regions are depicted on the diagram of the nervous system. L) Quantification of cells that express *P[GawB]4-57*-driven GFP in the presence and absence of Cha-Gal80, (n = 8, asterisk denotes p<.05). Scale bars = 50 µm.

Gal4 expression can be further refined using transgenic constructs that drive expression of the Gal4-inhibitor Gal80 [21]–[23]. Courtship assays using the Cha-Gal80 driver, which expresses in cholinergic neurons, combined with *P[GawB]4-57*(+) *UAS-fru^M^IR* transgenes revealed a significant rescue of the courtship defects (Figure 2B, G: green arrows). In *P[GawB]4-57*; *UAS-fru^M^IR*, *Cha-Gal80* males, courtship onset, courtship index, and copulation rates were similar to controls. These results should delimit the population of *fru^M^*(+) cells responsible for the courtship defects to only those in which the *Cha-Gal80* and *P[GawB]4-57* drivers were active (Figure 3).

### Fru^M^ Protein and *P[GawB]4-57* show Limited Overlap in the Nervous System

To assess the overlap of *P[GawB]4-57* and Fru^M^ protein, we first visualized the overlap of anti-Fru^M^ fluorescence with either *P[GawB]4-57*-driven *UAS-GFPnls* or *UAS-mCD8-GFP* (Figure 3A–E). Prime and double-prime symbols mark panels showing anti-Fru^M^ or merged anti-GFP+anti-Fru^M^ fluorescence respectively. Membrane tethered mCD8-GFP allowed us to visualize neuronal projections, while nuclear localized GFPnls allowed a direct comparison with the nuclear expression of Fru^M^.

*P[GawB]4-57*-driven GFP consistently overlapped with two Fru^M^(+) cells in cluster 7, approximately 6 in cluster 8 in the brain (Figure 3A–C) and two cells each in clusters 17 and 20 in the ventral nerve cord (Figure 3D–E), tabulated in Figure 3L based on 8 samples per genotype. Inconsistent overlap was seen in clusters 3 and 16.

Figure 3A′ shows two GFP(+), Fru^M^(+) cells in cluster 7, also known as mcAl [5]) or DT [13]) cells. In the SOG, overlap could be subdivided into three A–P subpopulations: four to six anterior, aSG; two medial, mSG; and two posterior, pSG (Figure 3A–C). Figure 3D–E shows overlap in two cells in cluster 17 at the prothoracic/metathoracic boundary (D) and in cluster 20, the abdominal ganglia (E). No peripheral expression was detected (data not shown).

At this point we will refer to Fru^M^(+) cells by nomenclature given in [13]. The *P[GawB]4-*57(+) cluster 7 cells appear to be the DT6 neurons described in that previous study. Also in that study, they characterized 8 subpopulations in the SOG (aSG1-8, pSG1-2). Some of the *P[GawB]4-*57(+) aSG neurons, characterized here, appear to be either the aSG5 or aSG6 cells. The *P[GawB]4-*57(+) mSG and pSG cells described here do not appear to confirm to either the pSG1 or pSG2 populations based on anatomy. A diagram of the nervous system showing Fru^M^ expression and cluster locations is shown in Figure 3K, arrows point to locations of the cells in each row of panels.

### *Cha-Gal80* Repressed *P[GawB]4-57*-driven GFP Primarily in DT6 and SOG Neurons

We noted that Cha-Gal80 repressed a significant fraction of the *P[GawB]4-57*-driven UAS-mCD8GFP expression pattern. In Figure 3F–J, hollow arrowheads represent Fru^M^(+) cells where we failed to detect *P[GawB]4-57*-driven GFP in combination with *Cha-Gal80. Cha-Gal80* repressed GFP fluorescence mainly in the two *P[GawB]4-*57(+) mSG and two-three of the aSG neurons (Figure 3G–H, hollow arrowheads). Conversely *Cha-Gal80* reduced but did not eliminate GFP fluorescence in *P[GawB]4-*57(+) DT6 cells (Figure 3F, see subpanels 1–2 for clarity). In pSG neurons (Figure 2H) and ventral nerve cord neurons (Figure 2I–J), GFP fluorescence appeared unaffected. Combined with Cha-Gal80 in behavioral studies, we inferred that some of the aSG and both mSG cells were primarily responsible for the courtship defects seen with *P[GawB]4-57*-driven constructs. We must note, however, that using Cha-Gal80-mediated repression of GFP may not fully reflect the relationship between *P[GawB]4-*57 expression and behavioral phenotype. Temporal differences between Cha-Gal80 and *P[GawB]4-*57 expressions or incomplete repression of GFP are caveats to this inference.

### *fru^FLP^*∩4-57 Neurons Project to the Tritocerebrum and Ventral Nerve Cord

**Figure 4:**
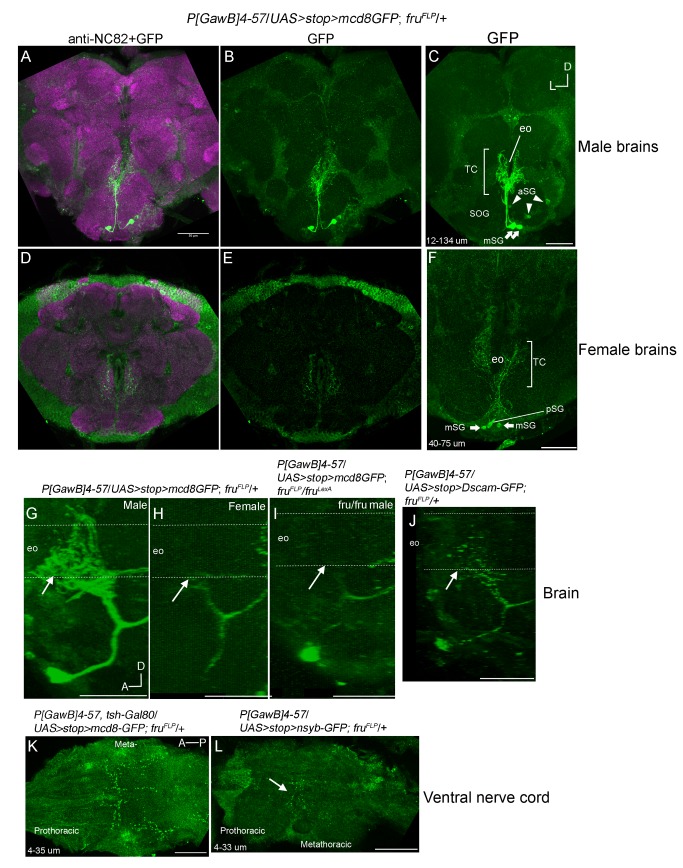
Visualization of *fru^M^*(+) and *P[GawB]4-57* intersection revealed a sexually dimorphic arborization in the tritocerebrum. A–F) Anterior-posterior, G–J) sagittal, and K–L) dorsal-ventral confocal projections. Unless otherwise stated, images are from males. A–I) Z-projections showing GFP fluorescence from male *P[GawB]4-57*/*UAS>stop>mCD8-GFP*; *fru^FLP^*/+ brains. A–F) Merged images showing anti-NC82 and GFP expressions in male and female brains. In all brains two GFP(+) cell bodies, in the ventral medial SOG (mSG, arrows) project to and make extensive arborizations in the tritocerebrum. C) In 7/14 brains, 3 cell bodies (aSG, arrowheads) fluoresced at depths of 17–20 µm without detectable neurites. D–F versus A–C) Z-projection showing the weaker tritocerebral aborizations from the two mSG neurons in female *P[GawB]4-57*/*UAS>stop>mCD8-GFP*; *fru^FLP^*/+ brains. F) pSG marks one posterior GFP(+) neuron. G–I), sagittal reconstructions of mSG projections in a G) *P[GawB]4-57*/*UAS>stop>mCD8-GFP*; *fru^FLP^*/+ male, H) female, and I) a *P[GawB]4-57*/*UAS>stop>mCD8-GFP*; *fru^FLP^*/*fru^LexA^* male mutant brain. Dashed lines mark the path of the esophagus. In *fru* ^+^/*fru* ^-^ males the tritocerebral arbors are significantly larger and fluoresce brighter compared to *fru* ^+^/*fru* ^-^ females or *fru* mutant males (arrows). J) Expression of the dendritic marker UAS>stop>Dscam17-1-GFP in mSG∩4-57 neurons colocalized with the tritocerebral arbors and anterior to medial tracts. L) Presynaptic marker, UAS>stop>nsyb-GFP was expressed mainly in prothoracic/metathoracic boundary. K) The presence of *tsh-Gal80* repressed expression from *fru^M^*(+) ventral nerve highlighting the descending projections from mSG and pSG cells. Scale bars = 50 µm. eo = esophageal foramen, TC = tritocerebrm, and SOG = subesophageal ganglion.

In order to highlight projection patterns only from *P[GawB]4-57*(*+*), Fru^M^(+) neurons, we visualized the GFP expression pattern in *P[GawB]4-57*/*UAS>stop>mCD8-GFP*; *fru^FLP^* animals, *fru^FLP^*∩4-57 (Figure 4). Fluorescence in *fru^FLP^*∩4-57 neurons revealed a smaller set compared to anti-Fru^M^:Gal4 overlap, labeling primarily the SOG neurons, with the mSG∩4-57 population the only consistent expression (Figure 4A–C).

**Figure 5:**
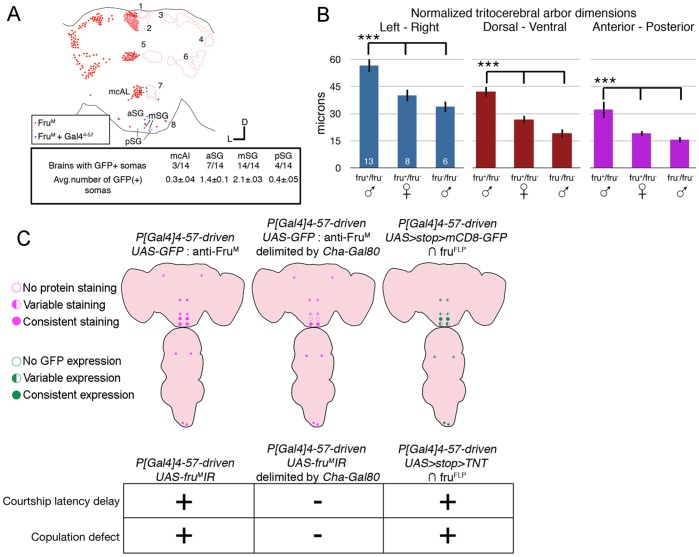
mSG∩4-57 expression correlated with behavioral defects. A) Proportion of brains exhibiting male *fru^FLP^*∩4-57 GFP expression (n = 14) and locations of GFP(+) neurons in *fru^M^* clusters (blue dots). B) Normalized (see methods) dimensions of the tritocerebral arbors measured in the lateral, D–V, and A–P axes for male *fru^M^*+/−, female *fru^M^*+/−, male fru^M^−/− mutant brains (n = 13, 8, 6). The tritocerebral arbors male *fru^M^* heterozygous brains were significantly larger than either male null *fru^M^* mutants or female *fru^M^* heterozygotes (p<.001). Diagram and table comparing *P[GawB]4-57*:Fru^FLP^ overlap, C) Cha-Gal80-delimited *P[GawB]4-*57:Fru^FLP^ overlap, *fru^FLP^*∩4-57 GFP expression, to RNAi-mediated or TNT-mediated behavioral phenotypes.

In 3/14 brains (Figure 5A), the aDT6∩4-57 neurons presented as one soma located in the periphery of the median bundle, with projections ramifying in the region proximal to the esophagus (eo), just dorsal to the tritocerebrum (Supplemental Figure 1S). Projections continued along the median bundle to the medial superior protocerebrum.

Most aSG∩4-57 GFP cells bodies in the SOG had no detectable projections (Figure 4C, arrowheads), but a single aSG∩4-57 neuron was seen, in two brains, with extensive arbors throughout the SOG with a collateral ramifying the inferior lateral protocerebrum (Supplemental Figure 1S, arrowhead, asterisks). pSG∩4-57 neurons sent projections to the ventral nerve cord with no detectable arbors in the brain.

Two mSG∩4-57 somas were found in the ventral medial SOG in 14/14 brains examined (Figure 4A–F, arrows). These exhibited similar bilateral projection patterns extending towards the tritocerebrum (TC) along with two descending tracts into the ventral nerve cord (Figure 4K–L).

### mSG∩4-57 Neurons make Sexually Dimorphic Projections in the Tritocerebrum

We noted a sexual dimorphism in the mSG projections. In female *fru^FLP^*/+ brains, fluorescence from the tritocerebral neurites was significantly reduced in extent and intensity compared to male projection patterns (compare Figure 4D–F to 4A–C). This difference was more striking in sagittal reconstructions of the tritocerebral projections (Figure 4G–H, arrows).

The behavioral defects caused by knockdown of *fru^M^* and the dimorphic projection pattern suggested a direct role for Fru^M^ in regulating neurite morphology in these neurons. In order to determine whether the sexual dimorphism required *fru^M^* expression, we examined the mSG∩4-57 projections in *fru^FLP^/fru^LexA^* mutant males (two different genetic backgrounds were used for the *fru^LexA^* chromosome). In these *fru* mutant males, the tritocerebral projections were similar to those seen in *fru^FLP^*/+ females (Figure 4I, arrow) indicating that *fru^M^* expression was required for proper male-specific arbors in the tritocerebrum.

### mSG∩4-57 Neurons make Descending Projections that Target the VNC

Descending tracts from the mSG∩4-57 and the pSG∩4-57 cells terminated as presynaptic arbors in prothoracic/mesothoracic (Figure 4K) and faintly in the mesothoracic/abdominal boundaries (not shown). We utilized the pre-synaptic marker nsyb-GFP and the somatodendritic marker Dscam17.1-GFP [13] to determine the neuronal polarity of these mSG∩4-57 neurons (Figure 4J, L). GFP fluorescence in *P[GawB]4-57*/*UAS>stop>Dscam17.1-GFP*; *fru^FLP^* brains were detected in the tritocerebral and anterior SOG tracts (Figure 4J). We detected expression of nsyb-GFP in *P[GawB]4-57*/*UAS>stop>nsyb-GFP*; *fru^FLP^*/+ brains in the prothoracic/mesothoracic boundary proximal to the likely positions of central pattern generators controlling wing song (Figure 4L). These data are consistent with a role for the mSG∩4-57 neurons in relaying signals targeting the tritocerebrum to the VNC and modulating targets within the ventral tritocerebrum. These were confirmed to be descending termini using the *tshirt-Gal80* transgene [13] to repress Gal4 activity in VNC *fru^M^*(+) neurons (Figure 4K).

Several lines of evidence combined to point to the significant role the subesophageal *P[GawB]4-57*(*+*), Fru^M^(+) likely play in regulating courtship initiation (Figure 5). One, rescue of the behavioral defects due to Cha-Gal80 repression mainly in the mSG neurons, (Figure 2B). Two, strong, consistent intersectional expression in mSG∩4-57 cells (Figure 5A). Three, sexual dimorphism of the tritocerebral arbors from the mSG∩4-57 cells (Figure 5B). Four, courtship and copulation defects seen upon *fru^FLP^*∩4-57 tetanus toxin expression (Figure 5C). Five, the behavioral defects were primarily seen under visually-deficient conditions (infrared) where male depend more on chemosensory cues, consistent with the putative function of tritocerebral dendritic projections of the mSG∩4-57 cells.

## Discussion

Initiation of unilateral wing extension is heavily dependent on visual, olfactory, and gustatory cues. By forcing males to depend on non-visual pathways for courtship and co-expressing tissue-specific *fru^M^* RNAi, we screened for *fru^M^*(+) neurons that likely regulate chemosensory-dependent processes in courtship, which manifested as infrared-specific courtship latency defects. The *P[GawB]4-57* line driving *UAS-fru^M^IR* possessed normal courtship latency in ambient light and significant infrared-specific delays. Notably *fru^M^* overlap was strongest in the SOG, while lacking any detectable peripheral expression. Behavioral and anatomical studies using Cha-Gal80, to subdivide the *P[GawB]4-57* expression pattern, highlighted a small subpopulation of *fru^M^*(+) neurons in the SOG, two-four anterior SG∩4-57 neurons and two medial SG∩4-57 neurons as responsible for the courtship defects.

### mSG∩4-57 Neuronal Anatomy Suggests a Direct Role in Regulating Wing Extension and Copulatory Behavior Initiation

**Figure 6:**
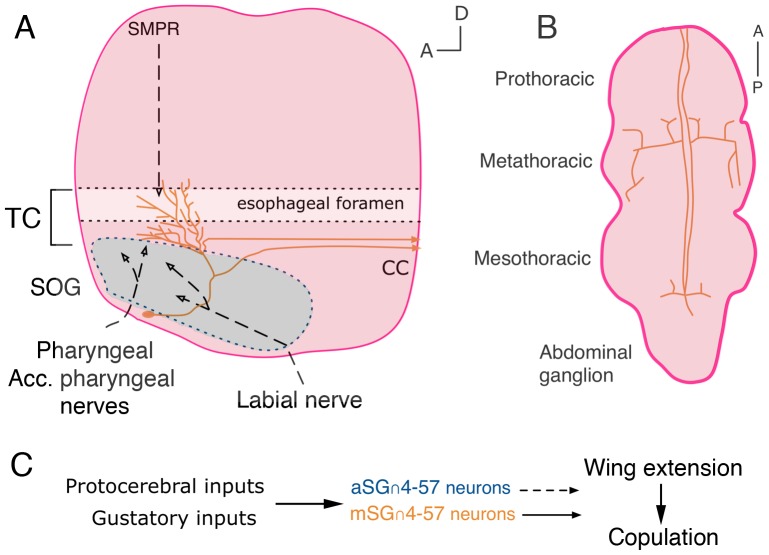
Projections *fru^M^* SOG∩4-57 neurons likely receive gustatory and descending protocerebral inputs. A) Sagittal views showing mSG∩4-57 neurons projections in the brain.. Gustatory inputs from the labellum, mouthparts innervate the anterior-medial SOG and the tritocerebrum. Inputs from the superior medial protocerebrum also innervate the tritocerebrum. Descending tracts from the mSG∩4-57 innervate prothoracic/metathoracic and mesothoracic/abdominal ganglia boundaries B). The mSG∩4-57, and possibly aSG∩4-57 neurons, function to regulate initiation of wing extension and copulatory behaviors. SMPR = superior medial protocerebrum, and CC = cervical connective. TC marks the tritocerebrum. The SOG is marked by a dashed blue line.

Several lines of evidence suggest a direct role for the mSG∩4-57 neurons in regulating the initiation of wing extension and copulatory behaviors. First, we detected expression of fluorescent markers in the mSG∩4-57 neurons driven by *P[GawB]4-57* in all brains, whereas fluorescence was only detected in a subset of animals for the other *fru^M^*∩4-57 subpopulations. The mSG∩4-57 neurons made sexually dimorphic arbors in the tritocerebrum (Figure 6A), where male arbors were significantly larger than in wild type female and *fru* mutant male brains. The mSG∩4-57 neuronal tracts extended into the VNC where presynaptic innervation of the mesothoracic triangle was seen (Figure 6B). The mesothoracic triangle is a target of descending command neurons that control wing song [15]–[17]. Faint projections were detected in the posterior metathoracic/anterior abdominal ganglia, which suggest possible regulation of motor circuitry needed for abdominal curling during copulatory behaviors.

The sexually dimorphic projections of the mSG∩4-57 suggest sex-specific roles in receiving tritocerebral signals in males. In males, *fru^M^* knockdown and silencing of *fru^FLP^*∩4-57 neurons resulted in a failure to progress to copulation, a behavior that follows proboscis contact with a female (“licking”). The internal mouthparts house gustatory sensilla that likely detect contact female pheromones accessed via licking behavior.

We cannot rule out functions for the non-mSG∩4-57 neurons, particularly the DT6∩4-57 (aSG) neurons (Figure 6C) in regulating courtship initiation, however. Our approach infers, but does not conclusively demonstrate that the mSG∩4-57 neurons are responsible for the courtship initiation and copulation defects. Further studies are required to conclusively identify the neurons responsible for each behavioral phenotype and their exact roles.

### A Comparison to *fru^M^*(+) SOG Neuron in other Studies

Several studies have examined the projections of *fru^M^*(+) neurons in the SOG. Antibody staining using anti-Fru^M^ identified 12±2 total Fru^M^(+) nuclei in the SOG in the 2-day pupal brain [5]. An intersectional study, using 131 Gal4 lines with sparse overlap with *fru^FLP^*, identified 8 *fru^FLP^*(+) SOG neuronal classes divided into six anterior, aSG1-6, and two posterior neuronal types, pSG1-2 [13]. At least one aSG∩4-57 neuron’s projection pattern, identified here, is consistent with the aSG5 class identified in that larger-scale study. Cachero *et al*. [14] used mosaic analyses of *fru^Gal4^* to identify larval neuroblast clonal populations of *fru^Gal4^* (+) neurons. Cachero *et al.* identified six clones in SOG, however, none appear to correspond to neurons identified here. It appears that these broad mapping studies, while extensive, have not exhaustively identified fru-expressing neurons in the SOG.

Using *tdc2-Gal4*, three studies [24]–[26] characterized three octopaminergic Fru^M^(+) neurons in the SOG: designated VPM1 and VPM2 (ventral paired median) and one VUM1 (ventral unpaired median) neuron. Expression of *tdc2-Gal4*-driven *UAS-fru^M^IR* leads to courtship latency delays but no copulation defect. The VUM1 neuron tritocerebral projections appear similar to the mSG∩4-57 projections, however, no descending tracts to the VNC were reported. The VPM1 and VPM2 appear to correspond to the DT8 neurons Repression of *fru^M^* using tdc2-Gal4 appeared to primarily disrupt male-female discrimination, resulting in significant male-male courtship, whereas we detected no significant male-male courtship using *P[GawB]4-57.*

### The Tritocerebrum is a Major Site of Signal Convergence for *fru^M^* Circuitry

Given the extensive projections of *fru^M^*(+) innervations, the tritocerebrum appears to be a site of gustatory integration with higher-order information in male courtship. The extensive, sexually dimorphic arbors from the mSG∩4-57 receive signals in the tritocerebrum that serve to regulate the progression to copulation in males and the performance of courtship. The tritocerebrum is targeted directly by gustatory afferents from the mouthparts via the pharyngeal nerves, indirectly via the SOG interneurons, which could relay signals from proboscis gustatory afferents entering via the labial nerve, and by descending tracts from the par interecerebralis of the superior medial protocerebrum (SMPR in Figure 6), which contains many neurosecretory cells [27]–[28]. These mSG∩4-57 cells could then relay signals to circuitry controlling wing extension/song in the metathoracic triangle and copulation/abdominal curling in the anterior abdominal ganglia.

The decision to perform courtship by males likely weighs the receptivity of the female versus the cost of female rejection via escape, with greater costs associated with later steps in the ritual, i.e. copulation. In open environs, escape behaviors exhibited by rejecting females likely results in the cessation of the courtship unless the male correctly gauges receptivity. We propose that the *fru^M^*(+) SOG neurons identified here play a vital link between detection of female receptivity cues and integration of higher-order signals in order to appropriate initiate wing extension and copulatory behaviors.

## Experimental Procedures

### Behavioral Assays

#### Courtship assays

Courtship assays were conducted according to established protocols [29]. Males were entrained in isolation for 3–5 days post-eclosion and then single males were presented with a 1–2-day-old Canton S virgin female. Single male and female pairs were placed into custom-made plexiglass chambers with hollowed circular chambers, 10 mm in diameter and 6 mm in height, separated by plastic transparencies. Contact between courtship pairs was initiated by removal of the transparencies. Courtship behaviors assayed in ambient fluorescent light and in infrared light, recorded for 10–15 minutes, and logged using the LifesongX annotation program. Initial screening of P[GawB] lines involved 9–12 males, while testing of candidate lines involved 20–40 males.

#### Activity monitoring

Recording of basal activity was done on according to established protocols [30]. Individual males, entrained and aged in the same method for courtship assays, were placed into glass tubes, sealed at one end and plugged with Drosophila media at the other end. The average number of line crossings, measured by an infrared laser, were recorded over a 24 hour period for 16–32 males using the Drosophila Activity Monitoring System I (TriKinetics).

### Fly Strains

*UAS-mCD8GFP* were obtained from the Bloomington Stock Center. *UAS-fru^M^IR*, *fru^P1-gal4^* and *fru^P1-LexA^* were constructed by D. Manoli [19], [21]. *fru^P1-LexA^* was backcrossed with >5 generations into the white Berlin background both the original stock and backcrossed strain was used. UAS>stop>mCD8-GFP, UAS>stop>TNT, UAS>stop>TNTQ4A, and fru^FLP^ lines are described in [13]. *P[GawB]* enhancer trap lines was obtained from Ulrike Heberliein [21].

### Constructs

LexAop-GFPnls: A BglII-SphI fragment containing a LexAop response element (a gift from D. Manoli) was swapped into pStinger [31] replacing the UAS element.

### Immunofluorescence

CNS and peripheral tissue were dissected and fixed using standard techniques [32]. Rat anti-Fru^M^ antibody was used at 1∶100 (Lee et al. 2000). Rabbit anti-GFP was used at 1∶500 (Invitrogen). CY3-conjugated goat anti-rat and FITC-conjugated goat anti-rabbit secondary antibodies were used at 1∶1000 (Jackson Immuno-research).

### Confocal Microscopy

Tissues were analyzed using a Zeiss LSM510 with a 40X oil objective. Images were taken at 1024×1024 pixels with slices at 1.0 to 1.5 µm intervals.

### Imaging Analyses

Image analyses were conducted using ImageJ and Fiji. The fluorescence density of the neurites was analyzed by sampling the integrated density of the target area and correcting for background by subtracting the fluorescence from three neighboring regions with no detectable labeled neurites. Normalization of tritocerebral arbors was achieved by establishing the average male, female, and fru mutant male brain size. Measurements were adjusted by a normalization factor per axis (sample brain axis/average brain axis).

### Statistical Analyses

JMP10 (SAS software) and R (http://www.r-project.org/) were used for statistical analyses.

#### Partial least squares

PLS does an iterative extraction on both the predictor (*P[GawB]*:*fru^P1-LexA^* overlap) and response (courtship latency) data sets to derive latent variables with the constraint that these latent variables explain the covariance between the data sets [33]. Significance testing was done using random resampling without replacement of the original overlap data set. 500 resampled matrices were constructed and analyzed with PLS to estimate confidence intervals.

#### Behavior analyses

ANOVA was conducted with a Tukey HSD post-test to determine significance of differences in courtship log-transformed latency means. For graphs, latency was back-transformed. Correlation of activity with latency was tested by Pearson’s correlation. Significance tests for courtship index and courtship/copulation percentages were done using Fisher’s exact test. Confidence intervals for proportions were estimated using the Clopper-Pearson interval.

## Supporting Information

Figure S1 **aSG∩4-57 projections.** Frontal confocal projections from *P[GawB]^4-57^*/*UAS>stop>mCD8-GFP*; *fru^FLP^*/+ animals. In this brain, a DT6∩4-57, aSG∩4-57, two mSG∩4-57, and one pSG∩4-57 neuron are visible. The DT6∩4-57 neuron projects to the superior medial protocerebrum (smpr). Extensive, fine arbors from the aSG∩4-57 neuron project bilaterally throughout the SOG. A collateral extends to the inferior lateral protocerebrum (ilpr). Not visible in this section the pSG∩4-57 neuron extends descending into the cervical connective. Scale bar = 50 µm.
(TIF)
