# Supplementary figures and images for: A Small Subset of Fruitless Subesophageal Neurons Modulate Early Courtship in Drosophila
Source: PLoS One. 2014 Apr 16;9(4):e95472. doi: 10.1371/journal.pone.0095472 (PMC3989346; doi:10.1371/journal.pone.0095472)

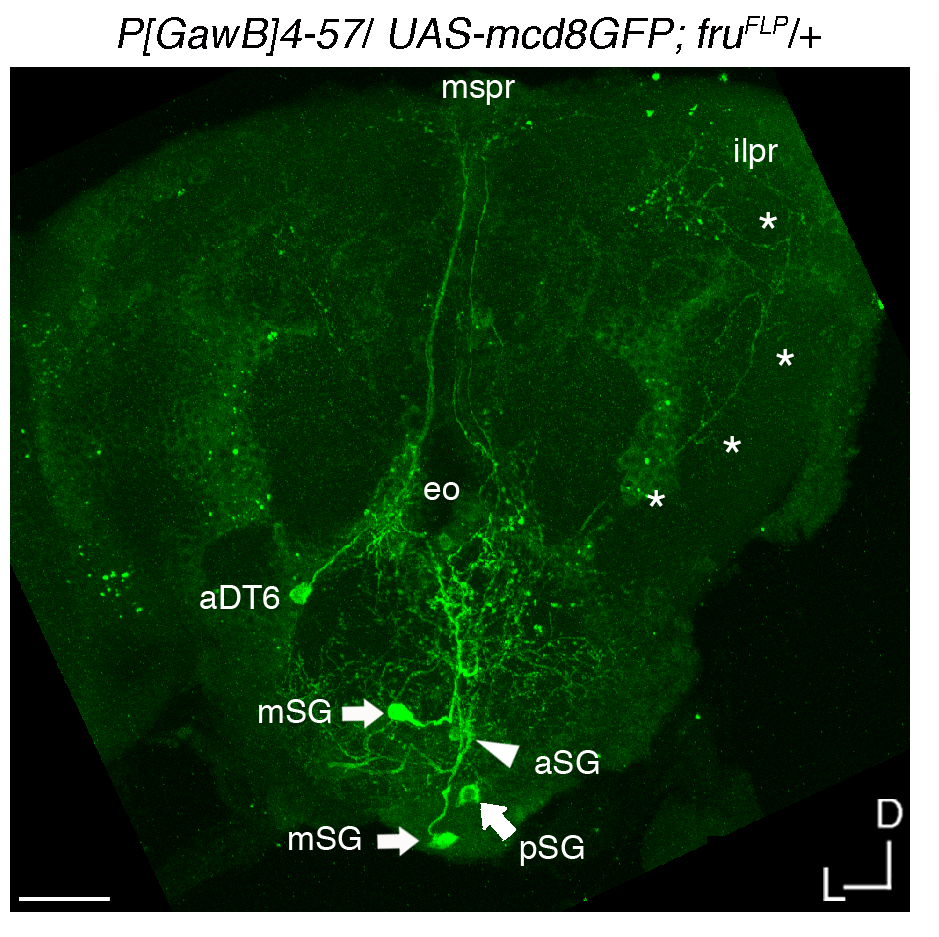

Supplement: Figure S1 — aSG∩4-57 projections. Frontal confocal projections from P[GawB]4-57/UAS>stop>mCD8-GFP; fruFLP/+ animals. In this brain, a DT6∩4-57, aSG∩4-57, two mSG∩4-57, and one pSG∩4-57 neuron are visible. The DT6∩4-57 neuron projects to the superior medial protocerebrum (smpr). Extensive, fine arbors from the aSG∩4-57 neuron project bilaterally throughout the SOG. A collateral extends to the inferior lateral protocerebrum (ilpr). Not visible in this section the pSG∩4-57 neuron extends descending into the cervical connective. Scale bar = 50 µm. (TIF) [file pone.0095472.s001.tif]
